# The comparative efficacies of intravenous administration and intra-articular injection of tranexamic acid during anterior cruciate ligament reconstruction for reducing postoperative hemarthrosis: a prospective randomized study

**DOI:** 10.1186/s12891-021-03990-7

**Published:** 2021-01-26

**Authors:** Rui Ma, Mengjun Wu, Yongwei Li, Jialin Wang, Wei Wang, Pei Yang, Kunzheng Wang

**Affiliations:** Department of Bone and Joint Surgery, the Second Affiliated Hospital of Xi’an Jiaotong University, Xi’an, 710004 Shaanxi China

**Keywords:** Tranexamic acid, Anterior cruciate ligament reconstruction, Hemarthrosis, Intravenous administration, Intra-articular injection

## Abstract

**Background:**

Hemarthrosis after anterior cruciate ligament (ACL) reconstruction can create many adverse joint effects. Tranexamic acid (TXA) can be used to minimize hemarthrosis and associated pain after ACL reconstruction. We aimed to compare the efficacies of intravenous (IV) administration and intra-articular (IA) injection of TXA during ACL reconstruction for reducing postoperative hemarthrosis.

**Methods:**

A total of 120 patients who underwent arthroscopic ACL reconstruction were included in this prospective and randomized study. All patients were randomized into three groups: IV group, IA group and placebo group. Patients in the IV group received intravenously administered TXA (15 mg/kg in 100 mL of saline solution) 10 min before tourniquet release; patients in the IA group received intra-articular TXA (15 mg/kg in 100 mL of saline solution) injected via the drainage tube; and patients in the placebo group received an equivalent volume of normal saline administered into the knee joint cavity and intravenously. Drainage tubes were removed 24 h after surgery, and all enrolled patients experienced a 4-week follow-up period. The drain output volume, visual analogue scale (VAS) score, patellar circumference, hemarthrosis grade and Lysholm score of all patients were recorded.

**Results:**

Both the IV group and the IA group had significantly lower drain output volumes at day 1, lower VAS scores at weeks 1 and 2, smaller patellar circumferences at weeks 1 and 2, and lower hemarthrosis grades at weeks 1 and 2 than the placebo group (*p* < 0.05). There were no significant differences in drain output volume, VAS score, patellar circumference or hemarthrosis grade between the IV group and the IA group at any time point (*p* > 0.05). No obvious differences in Lysholm score were observed between any pair of groups at week 4 (*p* > 0.05)). Neither infection nor deep vein thrombosis occurred in any group.

**Conclusions:**

Both intravenous administration and intra-articular injection can reduce intra-articular hemarthrosis, joint pain and swelling during ACL reconstruction. No significant difference in the efficacies of reducing hemarthrosis, joint pain and swelling was found between intravenous administration and intra-articular injection.

**Trial registration:**

The study was registered by the Chinese Clinical Trial Registry (The comparative efficacies of intravenous administration and intra-articular injection of tranexamic acid during anterior cruciate ligament reconstruction; ChiCTR-INR-17012217; August 1, 2017).

## Background

With the popularization of sports and the frequent occurrence of accidents, the incidence of anterior cruciate ligament (ACL) injury increases year by year [1]. Arthroscopically assisted ACL reconstruction (ACLR) is a common, reproducible and minimally invasive procedure. However, after ACLR, hemarthrosis and its associated pain afflict patients and can disrupt rehabilitation programs [2]. Postoperative hemarthrosis has a 3 to 10% incidence after ACLR [2]. Hemarthrosis creates many adverse effects, including increased susceptibility to infection, potential toxic effects to the cartilage, possible subsequent synovitis and arthrofibrosis, the onset of fever episodes, delayed rehabilitation, and prolonged hospital stay [2]. These aspects negatively affect the outcomes of patients and result in increased costs [3]. Minimizing postoperative intra-articular hemarthrosis may be potentially advantageous for recovery in ACLR patients.

Tranexamic acid (TXA), as an antifibrinolytic agent derived from lysine, binds to lysine receptor sites on plasminogen and prevents the formation of plasmin, thus inhibiting the breakdown of fibrin clots and reducing active bleeding [4]. TXA has a great role in reducing postoperative blood loss, the need for transfusion and hospital-related costs after knee surgery without increasing the surgical risks [5–8]. There are two major administration routes in the use of TXA: intravenous (IV) administration and intra-articular (IA) injection. Both IV administration and IA injection of TXA have been previously reported to effectively reduce hemarthrosis during ACLR. Some studies reported that IV administration of TXA could reduce the amount of drainage blood and postoperative hemarthrosis, reduce fever episodes and the need for aspiration of the knee, and improve knee range of motion (ROM) and early-phase outcomes without side effects after ACL reconstruction [9, 10]. Research on intra-articular injection of TXA during ACLR reported that it could significantly reduce postoperative intra-articular bleeding, pain and the grade of hemarthrosis, and no systemic side effects or need for aspiration was noted during the follow-up period [11]. However, the comparative efficacies of IV and IA TXA for reducing postoperative hemarthrosis during ACLR remain elusive.

This study aimed to compare the efficacies of intravenous administration and intra-articular injection of TXA during ACLR for improving early-phase outcomes. The hypotheses were that both IV administration and IA injection of TXA could reduce postoperative hemarthrosis, the amount of drainage blood, and pain, and that their performances might be similar.

## Methods

### Patients

**Fig. 1:**
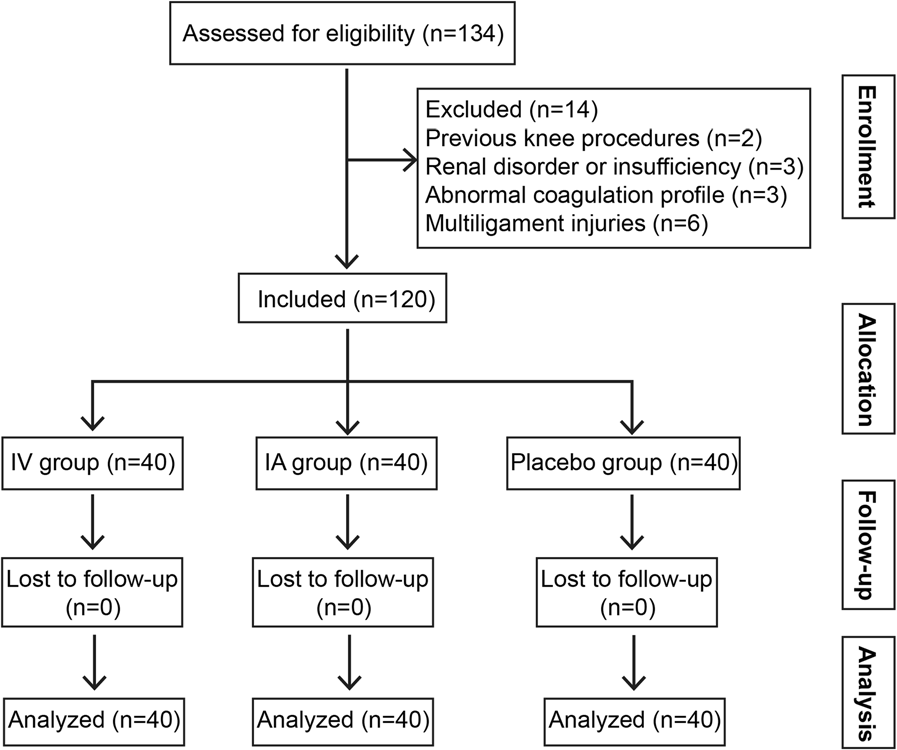
Patient flowchart

This was a single-center, prospective and randomized controlled study. Patients who underwent arthroscopic ACL reconstruction due to ACL rupture from December 2016 to December 2019 at our study location were enrolled consecutively. The exclusion criteria were as follows: previous knee procedures on the same side, renal disorder or insufficiency, abnormal coagulation profile, treatment with drugs interfering with coagulation or TXA clearance, multiple ligament injury, patients affected by blood, hemorrhagic or liver disease, and patients with nonsteroidal anti-inflammatory or antiplatelet therapy. This study was approved by the medical ethics committee of the Second Affiliated Hospital of Xi’an Jiaotong University. All participants gave written informed consent. Finally, 120 patients (74 males and 46 females) were included, with an average age of 31.0 ± 8.1 years (Fig. 1). The age, sex, body mass index (BMI) and time from injury to surgery of all patients were recorded.

### Surgery

Transtibial single-bundle reconstructions using autologous hamstring tendon grafts were performed in all patients; the graft was fixed using the RIGIDLOOP Adjustable Cortical System (DePuy Mitek, USA) for femoral fixation and MILAGRO Advance Interference Screws (DePuy Mitek) for tibial fixation. The intraoperative data registered were operative time, partial meniscectomy, meniscus repair, and tunnel diameter. General anesthesia or spinal/epidural block was selected according to the individual condition of each patient. The drainage tubes were clamped for 2 h before release. A pneumatic tourniquet was used in all patients and was released after skin closure. All operations were performed by the same team of surgeons. Cefazolin was administered 30 min before surgery for short-term prophylaxis. The analgesia regimen was intravenous administration of ketorolac (30 mg every 8 h) for the first day and oral administration of celecoxib (200 mg twice daily) for the next 5 days.

### Interventions

All patients were randomly divided into three groups (the intravenous group, intra-articular group and placebo group) by computer-generated randomization, with 40 patients in each group. Patients in the intravenous group received intravenously administered TXA (15 mg/kg in 100 mL of saline solution) 10 min before tourniquet release; patients in the intra-articular group received intra-articular TXA (15 mg/kg in 100 mL of saline solution) injected via the drainage tube; and patients in the placebo group received an equivalent volume of normal saline administered into the knee joint cavity and intravenously. The surgeon, anesthetist, patients and observer were blinded in regard of the use of tranexamic acid.

### Rehabilitation

A standard protocol was used for the rehabilitation program. Muscular training included exercises for the quadriceps, hamstrings and core stability at each stage. Patients were discharged with a long brace that was locked in the fully extended position at rest for 6 weeks postoperatively, and they were instructed to partially to fully bear weight with crutches under the protection of the fully extended brace according to their tolerance. After 4 weeks postoperatively, a full range of passive activity was allowed, with an increase in the flexion ROM of 15° per day up to ≥120°.

### Clinical evaluations

The drainage tubes were routinely removed, and the volume of drainage was recorded 24 h after surgery. All enrolled patients experienced a 4-week follow-up period. After 1, 2 and 4 weeks postoperatively, the visual analogue scale (VAS) score [12], patellar circumference, hemarthrosis grade [13] and Lysholm score [14] were recorded. The patellar circumference was measured 1 cm proximally to the superior border of the patella of both knees to evaluate joint swelling. To minimize interindividual variability in the patellar circumference, we considered the difference between the affected side and the contralateral side [9]. The clinical grading of hemarthrosis was scored according to Coupens and Yates [13]. Patients were accurately examined for the presence of leg swelling or calf pain, and those with suspected deep venous thrombosis (DVT) were recommended to undergo Doppler sonography. The postoperative evaluation was performed by a trained orthopaedic surgeon (R.M.) who was blinded to the treatment groups.

### Statistical analysis

The sample size was determined at a website (url: http://powerandsamplesize.com/Calculators/). In the sample size determination, multiple comparisons were made using the drain output volumes. The effect size, standard deviation, study power, and significance level were 75, 40, 0.80, and 0.05, respectively, and 39 patients were required in each group. All continuous variables are expressed as the mean ± SD. All data were managed through SPSS (IBM SPSS Statistics 19, USA). All continuous variables were analyzed by a Shapiro-Wilk test to evaluate the normal distribution of quantitative data. Indicators conforming to normal distribution were expressed in as mean ± standard deviation; a one-way analysis of variance (ANOVA) test was used for comparisons within groups, and least significant difference (LSD) test was used for comparison between each pair of groups. Indicators that did not conform to normal distribution were described as median and quartile; a Kruskal-Wallis H test was used for comparison within groups, and a Mann-Whitney U test was used for comparison between each pair of groups. The χ^2^ test or Fisher exact test was used to compare the categorical variables. Differences were considered significant at *p* < 0.05.

## Results

In this study, 120 patients were included and completed the follow-up, and 40 patients were included in each group. Table 1 shows the comparison of preoperative and intraoperative data in the IV group, IA group and placebo group. According to the demographic data, there were no significant differences in age, sex or BMI among the different groups (*p* = 0.272 for age; *p* = 0.781 for sex; *p* = 0.353 for BMI). When comparing the time from injury to surgery and the operation time, no statistically significant differences were found among the three groups (*p* = 0.590 for time from injury to surgery; *p* = 0.100 for operation time). The proportions of patients who received general anesthesia and spinal/epidural blocks in the different groups were similar (*p* = 0.590). According to the meniscus treatment, the surgical methods were ACLR alone, ACLR + partial meniscectomy and ACLR + meniscus repair. We found that there was no significant difference in the proportions of patients who underwent ACLR alone, ACLR + partial meniscectomy and ACLR + meniscus repair in the IV group, IA group and placebo group (*p* = 0.854). In addition, no significant difference was found among the three groups in terms of tunnel diameter (*p* = 0.281).

**Table 1:** Preoperative and intraoperative data

	IV group	IA group	Placebo group	F/χ^2^ value	*p* value
Age (years)	32.7 ± 8.5	30.3 ± 8.0	30.1 ± 7.7	1.318	0.272
Sex (Male/Female)	25/15	26/14	23/17	0.494	0.781
BMI (kg/m^2^)	22.1 ± 2.4	22.5 ± 2.5	22.9 ± 2.4	1.050	0.353
Time from injury to surgery (weeks)	25.5 ± 11.5	23.9 ± 11.8	22.9 ± 10.5	0.531	0.590
Operation time (minutes)	76.5 ± 11.0	71.3 ± 10.1	74.4 ± 11.8	2.344	0.100
General anesthesia, n. (%)	12 (30.0)	13 (32.5)	9 (22.5)	0.494	0.781
Spinal/epidural block, n. (%)	28 (70.0)	27 (67.5)	31 (77.5)		
ACLR alone, n. (%)	17 (42.5)	17 (42.5)	13 (32.5)	1.341	0.854
ACLR + partial meniscectomy, n. (%)	14 (35.0)	13 (32.5)	17 (42.5)		
ACLR + meniscus repair, n. (%)	9 (22.5)	10 (25.0)	10 (25.0)		
Tunnel diameter (cm)	8.13 ± 0.48	8.10 ± 0.40	8.15 ± 0.44	1.270	0.281

Table 2 shows the comparison of postoperative outcomes among the different groups. The drain output volumes of the IV group, IA group and placebo group were 78.5 ± 38.4 mL, 63.3 ± 31.8 mL and 120.1 ± 57.4 mL, respectively. Statistical analysis showed that the drain output volumes of the IV group and IA group were significantly higher than that of the placebo group (*p* = 0.000). Although the drain output volume of the IV group was slightly higher than that of the IA group, no significant difference was found between them (*p* = 0.123).

**Table 2:** Postoperative early-phase outcomes

	IV group	IA group	Placebo group	F/χ^2^ value	*p* value
Day 1					
Drain output volume (mL)	78.5 ± 38.4^c^	63.3 ± 31.8^c^	120.1 ± 57.4^a, b^	21.522	< 0.001
Week 1					
VAS score	2.7 ± 1.0^c^	2.4 ± 1.1^c^	3.5 ± 0.9^a, b^	12.793	< 0.001
Patellar circumference (cm)	18.1 ± 5.0^c^	19.4 ± 6.2^c^	24.6 ± 7.0^a, b^	12.549	< 0.001
Hemarthrosis grade, 0/1/2/3/4, n.	9/16/8/5/2^c^	8/16/11/4/1 ^c^	0/5/11/15/9^a, b^	35.221	< 0.001^*^
Week 2					
VAS score	2.3 ± 1.1^c^	2.2 ± 1.1^c^	3.1 ± 1.1^a, b^	8.343	< 0.001
Patellar circumference (cm)	11.2 ± 4.2^c^	10.0 ± 3.4^c^	16.0 ± 5.4^a, b^	20.446	< 0.001
Hemarthrosis grade, 0/1/2/3/4, n.	13/19/6/2/0^c^	13/18/8/1/0^c^	4/16/16/4/0^a, b^	13.186	0.031
Week 4					
VAS score	1.7 ± 1.2	1.7 ± 1.2	1.9 ± 1.4	0.265	0.768
Patellar circumference (cm)	5.7 ± 3.1	5.3 ± 3.2	5.8 ± 3.9	0.258	0.773
Hemarthrosis grade, 0/1/2/3/4, n.	16/17/5/2/0	18/17/2/3/0	11/18/8/3/0	5.742	0.455
Lysholm score	80.9 ± 11.8	82.1 ± 11.1	78.6 ± 10.6	1.026	0.362

^*^Fisher-Freeman-Halton exact test;

^a^Significant difference compared to the IV group (*p* < 0.05)

^b^Significant difference compared to the IA group (*p* < 0.05)

^c^Significant difference compared to the placebo group (*p* < 0.05)

The VAS scores and patellar circumferences of each group decreased gradually from 1 to 4 weeks postoperatively (Table 2). The VAS scores of the IV group and IA group were significantly lower than those of the placebo group 1 week and 2 weeks postoperatively (at 1 week: *p* = 0.000; at 2 weeks: IV vs. placebo *p* = 0.001, IA vs. placebo *p* = 0.000), but there was no significant difference in VAS scores between the IV group and IA group (at 1 week: *p* = 0.195; at 2 weeks: *p* = 0.680). The patellar circumferences of the IV group and IA group were significantly larger than those of the placebo group 1 week postoperatively (*p* = 0.000), but no significant difference was found between the IV group and IA group (*p* = 0.354); the comparison of the patellar circumference between different groups at 2 weeks after surgery had similar results. The hemarthrosis grades of the IV group and IA group at weeks 1 and 2 were significantly lower than that of the placebo group (at 1 week: *p* = 0.000; at 2 weeks: IV vs. placebo *p* = 0.015, IA vs. placebo *p* = 0.024), but the hemarthrosis grades of the IV group and IA group at weeks 1 and 2 were similar (at 1 week: *p* = 0.936; at 2 weeks: *p* = 0.932).

Four weeks postoperatively, the VAS scores, patellar circumferences and hemarthrosis grades of the IV group, IA group and placebo group exhibited no significant differences (*p* = 0.768 for VAS scores; *p* = 0.660 for patellar circumferences; *p* = 0.455 for hemarthrosis grades; Table 2). There were no infections in either group, and no patient developed DVT by postoperative week 4.

## Discussion

In this study, we found both intravenous administration and intra-articular injection can reduce intra-articular hemarthrosis, joint pain and swelling during ACL reconstruction, and no significant difference in the efficacies of reducing hemarthrosis, joint pain and swelling was found between intravenous administration and intra-articular injection.

Intravenous administration of TXA can reduce the amount of drainage blood and postoperative hemarthrosis after ACLR without side effects [9, 10, 15] (Table 3). In this study, it was found that intravenous administration of TXA could reduce postoperative drainage volume and hemarthrosis following ACLR and alleviate pain and knee swelling, similar to the results of the previous study [9, 10, 15]. TXA may penetrate large joints efficiently after IV administration [9, 16]. TXA was shown to exert its beneficial effects not only by reducing blood loss but also through its anti-inflammatory effects, which might improve analgesia after surgery [17]. This might explain the significantly lowered VAS score in patients who received IV-administered TXA. However, we found that IV administration of TXA did not significantly improve the Lysholm score at 4 weeks. Hetsroni [15] reported that compared to no use of TXA, IV administration of TXA did not improve the Lysholm score at 3 months in patients undergoing ACLR, but Karaaslan et al. [10] found a significant difference in the Lysholm score between IV administration and no use of TXA 2 and 4 weeks postoperatively. The differences in the comparisons of Lysholm scores might be attributed to differences in TXA dose and frequency in different studies. Because the mean duration of the effect of TXA is approximately 3 h, some studies suggested that a second dose should be administered to extend the effect over the first 6 h, when most bleeding occurs [18, 19]. Our study and Hetsroni’s study [15] only gave intravenously administered TXA before tourniquet release, which might not be sufficient to maximize the positive physiological effects of TXA; however, 10 mg/kg of intravenous TXA was given before surgery and an intravenous infusion of 10 mg/kg/h was continued for 3 h after the operation in Karaaslan’s study [10]. The regimen for intravenous administration of TXA requires further investigation.

**Table 3:** The main results of all the RCTs investigating the effectiveness of TXA after ACLR

Authors	Route	Main results
Felli et al. [9]	IV	IV of TXA administration reduced hemarthrosis and the amount of drainage blood.
Karaaslan et al. [10]	IV	IV of TXA reduced the amount of postoperative hemarthrosis and reduced pain.
Hetsroni et al. [15]	IV	IV of TXA reduced drained blood volume on postoperative day 1 and hemarthrosis up to postoperative day 15 but did not improve clinical outcomes at 3 months.
Chiang et al. [11]	IA	IA of TXA could significantly reduce postoperative intra-articular bleeding in the first 24 h, and decrease pain and the grade of hemarthrosis in the early postoperative period.

In this study, we found that intra-articular injection of TXA could also significantly reduce drainage blood volume and postoperative hemarthrosis and relieve pain and knee swelling. Intra-articular injection of TXA can be quickly absorbed locally; the physiological half-life of TXA in the joint cavity is approximately 3 h [20], thus local hemostasis can be achieved. To date, there has been only one study on intra-articular injection of TXA to reduce intra-articular hemarthrosis after ACLR [11], and the results of that study were similar to ours. The confusion of intra-articular injection of TXA after ACLR was that it was unclear whether TXA could harm the knee environment, particularly by potentially causing apoptosis of chondrocytes and tenocytes. Some studies have shown that a higher concentration of TXA might have a detrimental effect on animal or human chondrocytes in vitro [21, 22]. Parker et al. [23] showed that TXA (with concentrations up to 40 mg/mL) had no effect on the glycosaminoglycan content of human articular chondrocyte hydrogels after 6 h of exposure. Ambra et al. [24] found doses of TXA for clinical topical use did not demonstrate any cytotoxic effects on cartilage explants in a Yucatan mini pig model. It is worth noting that a post-arthroscopic knee is filled with some irrigation fluid and hemarthrosis, which might further lower the true concentration of TXA. The optimal dosage of intra-articular TXA still needs to be clarified in the future.

To the best of our knowledge, no study has compared the efficacies of intravenous administration and intra-articular injection of TXA in reducing postoperative hemarthrosis after ACLR. We found that there were no significant differences between intravenous administration and intra-articular injection of TXA in reducing postoperative blood drainage and hemarthrosis, alleviating pain and knee swelling, and improving knee function. Similarly, the comparative efficacies of IV administration and IA injection for reducing blood loss during total knee arthroplasty (TKA) are still controversial. Some studies reported that compared to IV administration, IA injection of TXA seemed to be more effective in terms of reducing blood loss and transfusion frequency [21, 22, 25]. In addition, some researchers concluded that there was no statistically significant difference in blood loss and transfusion rates between IA and IV use in TKA [26, 27]. Most likely, either IA administration or IV injection had its own advantage. For example, Sarzaeem et al. [28] reported that IA injection of TXA was more effective at decreasing postoperative drainage after TKA, but IV administration of TXA seemed to reduce the number of transfused units and the magnitude of the drop in hemoglobin more effectively. The argument for intravenous administration of TXA was that only a small portion of intravenously administered TXA reached the target surgical site, whereas intra-articular application of TXA could supply a higher concentration. The argument for intra-articular administration of high dose of TXA is the problem of potential toxicity to chondrocytes. Doses of TXA for clinical topical use did not demonstrate any cytotoxic effects on cartilage in vitro and in vivo [23, 24]. Therefore, it may be the surgeon’s preference as to what route of administration to use.

There were several limitations in this study. First, the follow-up period (4 weeks) was not long enough and there were not enough observation times points for the Lysholm scores. The first 3 months postoperatively is a significant period for preventing a delay in the rehabilitation program, so the exact long-term effect of TXA application on outcomes after ACLR could not be effectively analyzed in this study. Second, we only administered TXA intravenously 10 min before tourniquet release, which might not be sufficient to maximize the positive physiological effects of TXA. The regimen of intravenous administration of TXA requires further investigation.

## Conclusion

Both intravenous and intra-articular applications of TXA were effective and efficient for reducing postoperative blood drainage, intra-articular hemarthrosis, joint pain and swelling after arthroscopic ACL reconstruction. The efficacies of intravenous administration and intra-articular injection of TXA during ACL reconstruction for reducing postoperative hemarthrosis were similar.

## Data Availability

The datasets during and/or analyzed during the current study are available from the corresponding author on reasonable request.

## Abbreviations

ACL: Anterior cruciate ligament
TXA: Tranexamic acid
IV: Intravenous
IA: Intra-articular
VAS: Visual analogue scale
ACLR: ACL reconstruction
ROM: Range of motion
BMI: Body mass index
DVT: Deep venous thrombosis
ANOVA: Analysis of variance
LSD: Least significant difference
TKA: Total knee arthroplasty
